# Health-seeking behaviours by gender among adolescents in Soweto, South Africa

**DOI:** 10.3402/gha.v8.25670

**Published:** 2015-02-02

**Authors:** Kennedy Otwombe, Janan Dietrich, Fatima Laher, Stefanie Hornschuh, Busisiwe Nkala, Lucy Chimoyi, Angela Kaida, Glenda E. Gray, Cari L. Miller

**Affiliations:** 1 Perinatal HIV Research Unit, Faculty of Health Sciences, University of the Witwatersrand, Johannesburg, South Africa; 2 Canada–Africa Prevention Trials Network, The Ottawa Hospital General Campus, Ottawa, ON, Canada; 3 Faculty of Health Sciences, Simon Fraser University, Burnaby, BC, Canada

**Keywords:** health-seeking, adolescent health, health promotion, risk behaviours, South Africa

## Abstract

**Background:**

Adolescents are an important age-group for preventing disease and supporting health yet little is known about their health-seeking behaviours.

**Objective:**

We describe socio-demographic characteristics and health-seeking behaviours of adolescents in Soweto, South Africa, in order to broaden our understanding of their health needs.

**Design:**

The Botsha Bophelo Adolescent Health Study was an interviewer-administered cross-sectional survey of 830 adolescents (14–19 years) conducted in Soweto from 2010 to 2012. Health-seeking behaviours were defined as accessing medical services and/or being hospitalised in the 6 months prior to the survey. Chi-square analysis tested for associations between gender, other socio-demographic and behavioural characteristics, and health-seeking behaviours.

**Results:**

Of 830 adolescents, 57% were female, 50% were aged 17–19 years, 85% were enrolled in school, and 78% reported experiencing medium or high food insecurity. Males were more likely than females to report sexual debut (64% vs. 49%; *p*<0.0001) and illicit drug use (11% vs. 3%; *p*<0.0001). Approximately 27% (*n*=224) and 8% (*n*=65) reported seeking healthcare or being hospitalised respectively in the previous 6 months, with no significant differences by gender. Services were most commonly sought at medical clinics (75%), predominantly because of flu-like symptoms (32%), followed by concerns about HIV (10%). Compared to females, males were more likely to seek healthcare for condom breakage (8% vs. 2%; *p*=0.02). Relative to males, a significantly higher proportion of females desired general healthcare services (85% vs. 78%; *p*=0.0091), counselling (82% vs. 70%; *p*<0.0001), and reproductive health services (64% vs. 56%; *p*=0.02).

**Conclusions:**

A quarter of male and female adolescents accessed health services in the 6 months prior to the interview. Adolescents reported a gap between the availability and the need for general, reproductive, and counselling services. Integrated adolescent-friendly, school-based health services are recommended to bridge this gap.

According to the 2011 South African Census, about 10% (*n*=5,175,448) of the South African population are adolescents aged 15–19 years old (1). Adolescence is characterised by transition from dependence on adults and caregivers to independent decision-making and, typically, the initiation and experimentation with sexual, alcohol, and sometimes drug use behaviours. While these behaviours occur to some extent almost ubiquitously among adolescents globally, they can have long-term health consequences, especially in countries such as South Africa coping with the endemic presence of HIV (2). The National Youth Policy of South Africa emphasized the following as particular health challenges for adolescents living in South Africa: teenage pregnancy, maternal mortality, reproductive and sexual health, HIV/AIDS, and non-communicable diseases (3). Of all major causes of adolescent morbidity and mortality, HIV/AIDS is currently a major concern. Among young people, HIV/AIDS was the second leading cause of death globally in 2012, with adolescents in sub-Saharan Africa disproportionately affected (4).

Prior studies from Soweto in South Africa have identified high rates of unintended pregnancies among adolescents (5, 6), leading to sexual and reproductive health needs like medical abortions (5), antenatal care (7), and prevention and treatment for sexually transmitted infections (STIs) including HIV/AIDS (8). In 2005, approximately 35% of adolescents in South Africa reported unintended pregnancies (5), indicating the need for accessible adolescent-friendly sexual and health reproductive services. Previous studies have reported that other healthcare services accessed by adolescents in South Africa include oncology care (9) and counselling for violence and psychological abuse (10).

The National Adolescent-Friendly Clinic Initiative (NAFCI) has attempted to improve the response of the South African public sector to the special needs of adolescent health by introducing ‘adolescent-friendly’ clinic accreditation. NAFCI has worked with clinics to build capacity and improve acceptability and quality of services for adolescents, especially with regard to sexual and reproductive health (11). In 2006, NAFCI was adopted by the Department of Health in South Africa transforming it to Youth Friendly Services (YFS) which trains healthcare providers and accredits facilities for adolescent health services. In the new YFS services, responsibility for media campaigns was not retained but is being championed by other non-governmental organisations like LoveLife that runs multimedia campaigns, peer outreach, youth centres, and peer education programmes as part of their HIV prevention campaign (12).

Research on health-seeking behaviours among adolescents in South Africa is limited. To help address this gap, we describe here the health-seeking behaviours of adolescents, by gender, within the peri-urban community of Soweto, South Africa.

## Methods

### Study design and setting

The Botsha Bophelo Adolescent Health Study (BBAHS) was an interviewer-administered cross-sectional survey of 830 adolescents conducted at the Kganya Motsha Adolescent Centre (KMAC) and the Perinatal HIV Research Unit (PHRU) in Soweto, South Africa, between June 2010 and June 2012. The BBAHS was a collaboration between Simon Fraser University in British Columbia, Canada, and the PHRU located in Soweto, South Africa.

KMAC was founded in 2008 targeting in and out-of-school adolescents and was among the first health clinics in South Africa mandated solely for adolescents and designed to provide adolescent-friendly healthcare services. It offered HIV voluntary counselling and testing (VCT) and sexual and reproductive healthcare services for adolescents aged between 14 and 19 years at no cost.

Soweto is a township in the south of Johannesburg with a population of approximately 1.3 million inhabitants (13). In the most recent census, there were 315,116 adolescents aged between 15 and 19 years in the City of Johannesburg metropolitan municipality (14).

### Participants

Participants were recruited through a stratified sampling approach across all regions (locally known as townships) that comprise Soweto. The sampling strategy was developed to target more females than males, those of older age (17–19 years) to include a higher proportion of sexually active adolescents and to ensure representation from all geographic locations within Soweto including those from formal and informal housing. Recruitment was conducted in 44 identified townships and at KMAC. A minimum of 15 adolescents were recruited from each township. Only those between 14 and 19 years and living in Soweto were eligible for enrolment. To ensure representation and inclusion of the hard-to-reach and out-of-school population within townships, recruitment occurred in a variety of locations such as community centres, shopping centres, and areas around schools. Participants were approached and informed about the study. Those willing to participate provided contact details for scheduling interview appointments. Interviews were completed at KMAC or PHRU, whichever was more convenient for individual participants.

To avoid enrolling participants more than once, identification was required for enrolment and recorded in a separate file. Each participant was then given a unique identifier code containing no personal information in order to maintain confidentiality.

### Data collection

Interviewers administered an online survey with participants. It assessed socio-demographics, sexual and reproductive health, HIV testing history, substance use (drugs and alcohol), and depressive symptoms. Trained, multilingual interviewers administered the survey in English or Isizulu (a local language in Soweto) via an online internet platform, Survey Monkey, using iPads or computers (Survey Monkey, 2012). The survey took an average of 60 min to complete.

### Measures

Socio-demographic measures included gender, age (≤15, 15–17 and 17–19 years), type of housing (living in a shack or other [house or apartment]), employment (yes vs. no), current enrolment in school (yes vs. no), whether parents were alive (yes vs. no), level of education (up to primary vs. high school or higher), and food insecurity.

The nine-item Household Food Insecurity Access Scale was used to measure food access within a 4-week time period (Cronbach alpha=0.82). Possible responses were ‘never’, ‘rarely’, ‘sometimes,’ and ‘often’. A score of 3 or more was considered a reflection of medium or high food insecurity with scores of 0–9, and higher scores indicating greater food insecurity (15).

Risk behaviours were assessed by asking whether the participant had ever had a STI, alcohol in the previous 6 months, and ever used drugs. Adolescents were asked if they had ever had sex (yes vs. no), had ever tested for HIV (yes vs. no), and their sexual orientation (heterosexual vs. non-heterosexual).

Depression was measured with the 20-point Center for Epidemiologic Studies Depression (CES-D) 9.3 scale (Cronbach alpha=0.81). It is used for the assessment of depressive symptomatology in the general population (16). Individual items are scored between 0 and 3, with the scale score ranging from 0 to 60. Based on previous evaluations of the CES-D among adolescent populations, we used a score ≥24 to determine the presence of depressive symptoms (17).

The primary outcome variable, health-seeking behaviour, was defined as having responded ‘Yes’ to the question, ‘Have you gone for medical/healthcare in the past 6 months?’. To assess where adolescents sought healthcare services and for what reasons, we asked; ‘Where did you go for medical/healthcare in the past 6 months?’ and ‘What was the reason you required medical/healthcare in the past 6 months?’. To determine hospital service use we asked; ‘In the past 6 months, have you been hospitalised for any reason?’, and ‘What was the reason for hospitalisation?’. Additional questions about health-seeking behaviours were, ‘Have you ever tested for HIV?’; and to determine the potential for gaps in service needs, we asked; ‘What social and health services would you or people you know like to have that you cannot currently find?’.

### Ethical considerations

Ethical approval to conduct the study was given by the ethics committees of the University of the Witwatersrand, Johannesburg, South Africa, and Simon Fraser University, Burnaby, Canada. The study was conducted in accordance with the Helsinki Declaration of 1975 as revised in 2008. Participants received 50 ZAR (~$7) for travel reimbursement. Written informed consent was obtained for all participants. Participants younger than 18 years required written parental consent with participant assent.

### Statistical analysis

Overall frequencies and frequencies by gender were determined for categorical socio-demographic and behavioural measures such as age-group and ever testing for HIV. Reliability of the assessment scales CES-D and food insecurity were assessed using the Cronbach alpha statistic.

Frequencies and percentages overall and by gender were determined for the items ‘Have you gone for medical/healthcare in the past 6 months?’, ‘Where did you go for medical/healthcare in the past 6 months?’, ‘What was the reason you required medical/healthcare in the past 6 months?’, ‘What social and health services would you or people you know like to have that you cannot currently find?’, ‘In the past 6 months, have you been hospitalised for any reason?’, and ‘What was the reason for hospitalisation?’. Where applicable, all items with the response ‘Other’ and ‘Please specify’ were categorised to fit in with the pre-specified categories or plausible medical classifications. Chi-squared tests were used to investigate associations between variables. ‘Other (please specify)’ responses to the item ‘What social and health services would you or people you know like to have that you cannot currently find?’ were assessed descriptively.

Where missing values occurred, we performed a complete case analysis. All statistical tests were performed at a 5% level of significance in SAS Enterprise Guide 5.1 (18).

## Results

### Participant characteristics

Socio-demographic and behavioural characteristics are presented in Table 1. A total of 830 adolescents, aged 14–19 years, enrolled in BBAHS, and there were more females than males (475 [57%] vs. 355 [43%]). The median age was 17 (IQR: 16–18) years. Most were attending high school (85.4%, *n*=709), and females were more likely than males to be in school (89.7% vs. 79.7%; *p*<0.0001). A total of 14.7% (*n*=122) of the adolescent participants lived in shacks, 7.6% (31/408) were employed and 91.7% (*n*=396) had at least one living parent. The proportion of adolescents stating medium-to-high food insecurity was 78.1%, with males more likely than females to be food insecure (81.6% vs. 75.5%; *p*=0.0339).

**Table 1:** Socio-demographic and behavioural characteristics of adolescents

Variable	Overall (*N*=830)	Males (*N*=355)	Female (*N*=475)	*p*
Age-group (years)				
≤ 15 (%)	179 (21.8)	86 (24.7)	93 (19.7)	0.2196
15–17 (%)	233 (28.4)	97 (27.9)	136 (28.8)	
17–19 (%)	408 (49.8)	165 (47.4)	243 (51.5)	
Education				
Up to primary (%)	121 (14.6)	72 (20.3)	49 (10.3)	**<0.0001**
High school (%)	709 (85.4)	283 (79.7)	426 (89.7)	
Do you live in a shack?				
Yes (%)	122 (14.7)	51 (14.4)	71 (14.9)	0.8150
No (%)	708 (85.3)	304 (85.6)	404 (85.1)	
Employment				
Yes (%)	31 (3.8)	18 (5.1)	13 (2.8)	0.0822
No (%)	794 (96.2)	336 (94.9)	458 (97.2)	
Parents alive				
Yes (%)	761 (91.7)	323 (91.0)	438 (92.2)	0.5272
No (%)	69 (8.3)	32 (9.0)	37 (7.8)	
Have you ever tested for HIV?				
Yes (%)	378 (47.0)	148 (43.5)	230 (49.6)	0.0901
No (%)	426 (53.0)	192 (56.5)	234 (50.4)	
Ever had an STI				
Yes (%)	20 (2.4)	12 (3.4)	8 (1.7)	0.1149
No (%)	810 (97.6)	343 (96.6)	467 (98.3)	
Ever had sex				
Yes (%)	461 (55.5)	228 (64.2)	233 (49.1)	**<0.0001**
No (%)	369 (44.5)	127 (35.8)	242 (50.9)	
Sexual orientation				
Heterosexual (%)	686 (82.7)	302 (85.1)	384 (80.8)	0.1115
Non-heterosexual (%)	144 (17.3)	53 (14.9)	91 (19.2)	
Had alcohol in the last 6 months				
Yes (%)	525 (64.1)	234 (66.9)	291 (62.0)	0.1557
No (%)	294 (35.9)	116 (33.1)	178 (38.0)	
Ever used drugs				
Yes (%)	53 (6.4)	38 (10.7)	15 (3.2)	**<0.0001**
No (%)	777 (93.6)	317 (89.3)	460 (96.8)	
Depression				
High (%)	399 (48.1)	159 (44.8)	240 (50.5)	0.1503
Low (%)	431 (51.9)	196 (55.2)	235 (49.5)	
Food insecurity				
Low (%)	181 (21.9)	65 (18.4)	116 (24.5)	**0.0339**
Medium/high (%)	646 (78.1)	289 (81.6)	357 (75.5)	
Have you gone for medical/healthcare in the past 6 months?				
Yes (%)	224 (27.0)	91 (25.6)	133 (28.0)	0.4474
No (%)	606 (73.0)	264 (74.4)	342 (72.0)	
In the past 6 months, have you been hospitalised for any reason?				
Yes (%)	65 (7.8)	35 (9.9)	30 (6.3)	0.0601
No (%)	765 (92.2)	320 (90.1)	445 (93.7)	

All bold values refer to variables that are statistically significant.

Almost half (47%, *n*=378) of the adolescents reported ever testing for HIV, and 2.4% (*n*=20) reported ever having an STI. Though 55.5% (*n*=461) of participants reported sexual debut, males were more likely to report this than females (64.2% vs. 49.1%; *p*<0.0001). Although the proportion of males and females reporting alcohol use was similar (66.9% vs. 62.0%; *p*=0.1557), more males than females reported drug use (10.7% vs. 3.2%; *p*<0.0001). A high score of depressive symptomatology was reported in 48.1% of adolescents and was similar between males and females.

### Characteristics of health-seeking behaviours

**Fig. 1:**
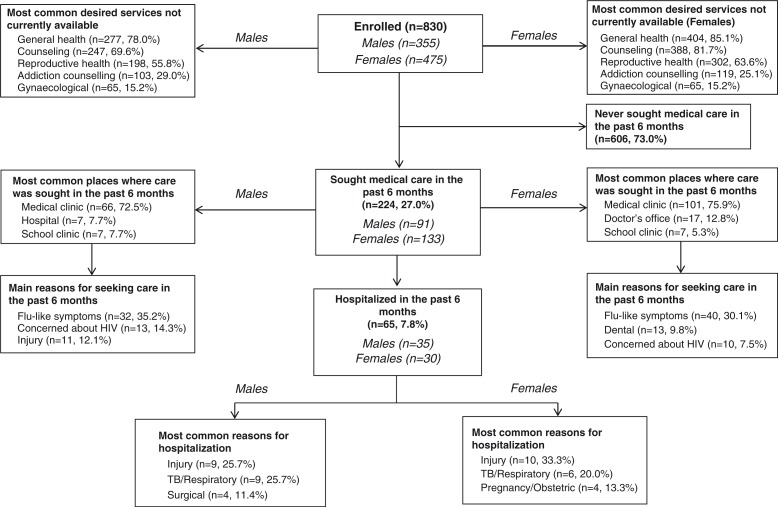
Flow chart of health-seeking behaviour and reasons among adolescents in Soweto, South Africa.

Figure 1 shows the most commonly desired services among adolescents. These were general health (85% among females and 78% among males), counselling (82% among females and 70% among males), reproductive health (64% among females and 56% among males), and addiction counselling (25% among females and 29% among males). Slightly more than one-quarter of participants had accessed medical care in the past 6 months with the majority seeking care through medical clinics (76% of females and 73% of males). The main reason for seeking medical care was flu-like symptoms (30% among females and 35% among males). Almost 8% of adolescents had been hospitalised in the previous 6 months, and the most common reason was injury (33% among females and 26% among males).

In Table 2, the location and reasons for adolescent health-seeking behaviour in the past 6 months are shown. Overall, 27.0% (224/830) of the adolescents reported seeking medical care in the previous 6 months, and the proportions by gender were similar (25.6% vs. 28.0%; *p*=0.4474). Healthcare was most commonly sought at local medical clinics (74.6%), followed by doctor’s practice (8.5%) and hospitals (6.3%). The proportion of female adolescents seeking healthcare in the doctor’s practice was significantly higher than for males (12.8% vs. 2.2%; *p*=0.0052). Approximately 5.4% of adolescents sought healthcare through a school clinic, and only 3.1% sought care through a family planning clinic. Overall, approximately 7.6% sought care through a homeopath and/or a traditional healer.

**Table 2:** Location and reasons for adolescent health-seeking behaviour in the past 6 months

Variable	Overall	Male	Female	p*
Have you gone for medical/healthcare in the past 6 months?	(*n*=830,%)	(*n*=355,%)	(*n*=475,%)	
Yes	224 (27.0)	91 (25.6)	133 (28.0)	0.4474
No	606 (73.0)	264 (74.4)	342 (72.0)	
Where did you go for medical/healthcare in the past 6 months?	(*n*=224,%)	(*n*=91,%)	(*n*=133,%)	
Medical clinic	167 (74.6)	66 (72.5)	101 (75.9)	0.5647
Doctor’s office	19 (8.5)	2 (2.2)	17 (12.8)	**0.0052**
Hospital	14 (6.3)	7 (7.7)	7 (5.3)	0.4607
School clinic	12 (5.4)	7 (7.7)	5 (3.8)	0.1992
Family planning/abortion clinic	7 (3.1)	5 (5.5)	2 (1.5)	0.0918
Kganya Motsha adolescent-friendly facility	5 (2.2)	3 (3.3)	2 (1.5)	0.3723
Other: homeopath (*n*=1), traditional/faith healer (*n*=1), not stated (*n*=15)	17 (7.6)	9 (9.9)	8 (6.0)	0.2821
What was the reason you required medical/healthcare in the past 6 months?	(*n*=224,%)	(*n*=91,%)	(*n*=133,%)	
Flu-like symptoms	72 (32.1)	32 (35.2)	40 (30.1)	0.4231
Concerned about HIV	23 (10.3)	13 (14.3)	10 (7.5)	0.1013
Injury	19 (8.5)	11 (12.1)	8 (6.0)	0.1091
Dental	16 (7.1)	3 (3.3)	13 (9.8)	0.0645
Birth control	11 (4.9)	3 (3.3)	8 (6.0)	0.3551
Condom broke	9 (4.0)	7 (7.7)	2 (1.5)	**0.0205**
Circumcision	9 (4.0)	8 (8.8)	1 (0.8)	–
Depression/suicidal thoughts	8 (3.6)	3 (3.3)	5 (3.8)	0.8546
Pregnancy test	8 (3.6)	1 (1.1)	7 (5.3)	–
Rash	7 (3.1)	1 (1.1)	6 (4.5)	–
Headache/other pain	6 (2.7)	0 (0)	6 (4.5)	–
Allergies/asthma	5 (2.2)	3 (3.3)	2 (1.5)	0.3723
Gastrointestinal illness	4 (1.8)	1 (1.1)	3 (2.3)	–
Abortion	3 (1.3)	1 (1.1)	2 (1.5)	–
Concerned about sexually transmitted infections	2 (0.8)	2 (2.2)	0 (0)	–
Concerned about tuberculosis	2 (0.8)	2 (2.2)	0 (0)	–
Other: Not stated (*n*=5), concern about breast cancer (*n*=2), eye illness (*n*=5), renal (*n*=1), surgical (*n*=3), routine check-up (*n*=2), epilepsy (*n*=1), menstrual problems (*n*=3), alcohol (*n*=1)	23 (10.3)	4 (4.4)	19 (14.3)	**0.0166**

Note: Respondents could check more than one response.

* *p-*Value comparisons compare the proportion of males and females.

All bold values refer to variables that are statistically significant.

The most common reason for seeking healthcare was flu-like symptoms (32.1%) followed by concerns about HIV (10.3%), injuries (8.5%), and dental problems (7.1%). Compared to females, males were more likely to seek care for condom breakage (7.7% vs. 1.5%; *p*=0.0205). Overall 4.9% of adolescents sought healthcare for birth control and 3.6% for depression and/or suicidal thoughts. Approximately 8.8% of the young male participants sought healthcare for circumcision-related reasons.

A summary of desired health services is presented in Table 3. The most common social and health services desired were general health services (e.g. flu-like symptoms; 82.0%), psycho-social counselling (e.g. for sexual abuse, violence, family; 76.5%), reproductive health services (e.g. contraception, pregnancy care, STIs; 60.2%), addictions counselling (drugs and alcohol; 26.7%), and gynaecological services (e.g. Pap smears; 19.9%). Relative to males, significantly more females desired general health (85.1% vs. 78.0%; *p*=0.0091), counselling (81.7% vs.69.6%; *p*<0.0001), and reproductive (63.6% vs.55.8%; *p*=0.0230) health services. Both females and males desired gynaecological services (20.2 and 19.4%; *p*=0.7822). About 5.9% (*n*=49) of the participants raised the point that they desired an improvement of current services with a focus on quality, friendliness, cleanliness, as well as avoiding medication and staff shortages (data not shown). In the 6 months prior to the interview, 7.8% (*n*=65) were hospitalised. The proportion by gender was borderline significant (9.9% for males vs. 6.3% for females; *p*=0.0601). Of those hospitalised, the most common reasons for hospitalisation were injuries (29.2%) followed by tuberculosis or respiratory illnesses (23.1%), surgical reasons (9.2%), and pregnancy or obstetric issues (6.2%).

**Table 3:** Desired health services and reasons for hospitalisation in the past 6 months

Variable	Overall	Male	Female	p*
What social and health services would you or people you know like to have that you cannot currently find?	(*n*=830,%)	(*n*=355,%)	(*n*=475,%)	
General health services (flu, etc.)	681 (82.0)	277 (78.0)	404 (85.1)	**0.0091**
Counselling (for sexual abuse, violence, family, etc.)	635 (76.5)	247 (69.6)	388 (81.7)	**<0.0001**
Reproductive health services (birth control, pregnancy, sexually transmitted infections, etc.)	500 (60.2)	198 (55.8)	302 (63.6)	**0.0230**
Addictions counselling (drugs, alcohol, etc.)	222 (26.7)	103 (29.0)	119 (25.1)	0.2021
Gynaecological services (pap smears, etc.)	165 (19.9)	69 (19.4)	96 (20.2)	0.7822
Improved service	49 (5.9)	14 (3.9)	35 (7.4)	**0.0383**
Abortion	43 (5.2)	23 (6.5)	20 (4.2)	0.1446
Tuberculosis	33 (4.0)	17 (4.8)	16 (3.4)	0.3002
Referral to circumcision	26 (3.1)	12 (3.4)	14 (2.9)	0.7232
Other: cancer screening (*n*=1), free food service (*n*=2), dental service (*n*=4), not stated (*n*=2), HIV/AIDS related (*n*=1)	10 (1.2)	4 (1.1)	6 (1.3)	0.8586
In the past 6 months, have you been hospitalised for any reason?				
Yes	65 (7.8)	35 (9.9)	30 (6.3)	0.0601
No	765 (92.2)	320 (90.1)	445 (93.7)	
What was the reason for hospitalisation?	(*n*=65,%)	(*n*=35,%)	(*n*=30,%)	
Injury	19 (29.2)	9 (25.7)	10 (33.3)	0.5008
Tuberculosis/respiratory	15 (23.1)	9 (25.7)	6 (20.0)	0.5857
Surgical	6 (9.2)	4 (11.4)	2 (6.7)	0.5085
Pregnancy/obstetric	4 (6.2)	0 (0)	4 (13.3)	–
Rape	3 (4.6)	3 (8.6)	0 (0)	–
AIDS related illness	2 (4.6)	1 (2.9)	1 (3.3)	–
Other: Overdose of alcohol (*n*=1), malaria (*n*=1), meningitis/headache (*n*=2), attempted suicide (*n*=2), menstrual problems (*n*=1), stomach ache (*n*=1), unclear (*n*=3)	11 (16.9)	5 (14.3)	6 (20.0)	0.5402

Note: Respondents could check more than one response.

* *p-*Value comparisons compare the proportion of males and females.

All bold values refer to variables that are statistically significant.

## Discussion

This study adds to the literature on health-seeking behaviours of adolescents. The primary strength of the study is that, unlike many other adolescent studies, it did not restrict recruitment to a type of venue, such as schools or clinics, but rather obtained the sample from the general population, thereby maximising the potential of the study to include vulnerable adolescents who may be outside of formal systems.

Our findings show that about one-quarter of adolescents sought healthcare services in the 6 months prior to the interview. Uptake appeared much higher in a school-based study in the US, where in their senior year, 71% of participants had visited a healthcare provider, 44% visited a school health centre, 41% visited a private doctor’s office, and 26% visited an emergency room (19). The differences in uptake may be explained by poorer economic circumstances in our participants who access the private sector infrequently, and the lack of school health centres in public sector South African schools. It is known that adolescents prefer to be separated from adult service provision (20), and the absence of school health services may indeed limit the ability to reach adolescents for health promotion.

In our study, the conventional public healthcare sector was the predominant entry point for adolescent care. In this sector, although general health, counselling, and reproductive health services do currently exist, adolescents perceived that these very services were unavailable to adequately meet their needs. This disconnection deserves further consideration. It is possible that adolescents were not aware of these services being present in their area. However, some adolescents noted barriers to care including perceived sub-optimal service quality. Adolescents’ desire for existing services to be improved and their ideas for improvement are consistent with findings from a recent audit of healthcare facilities in South Africa commissioned by the Department of Health (21). In that audit, only 30% of facilities were found to be compliant with positive and caring attitudes, 50% with cleanliness and 54% with availability of medicines and supplies. Taken together, these realities may discourage adolescents from seeking healthcare. One study showed that if clinics adhere to NAFCI standards, the quality of adolescent-friendly services could be significantly improved. However, NAFCI adherence on its own is insufficient: the health system should include adolescent voices in determining their own health needs, should educate them about health rights and inform them about service availability (11).

While the proportion of female adolescents seeking healthcare in our study was slightly higher than that of males, they were not significantly different, suggesting that health systems are a viable entry point for health promotion for both male and female adolescents. Other African studies have found gender disparity in adolescent health-seeking behaviours, with significantly more females accessing healthcare than males (22–24). In our study, the differences in service utilisation were not by gender, but rather by sexual activity and by HIV testing uptake. This suggests that health service utilisation for both male and female adolescents may follow from the initiation of sexual activity. Again it highlights the opportunity that health facilities have to promote sexual and reproductive health to male and female adolescents.

In our setting, the most common reason for seeking healthcare was flu-like symptoms, and this did not differ by gender. This finding is different to that of a recent multi-country study across Africa, North America and Asia, which, unlike our study, had a qualitative design. It found that reasons for seeking healthcare differed by gender: female adolescents sought healthcare for sexual and reproductive health reasons while males sought healthcare for drug and alcohol related reasons (24).

Adolescents indicated the need for more accessible counselling services, especially related to violence, family, and drug addictions. This is underscored by the high observed prevalence of reported depression symptoms. Previous research from South Africa demonstrates the high prevalence of substance use and inter-personal violence (10, 25, 26). While older reports from South Africa had found that epilepsy and pneumonia were the leading causes of adolescent hospitalisation (27), injuries were the leading cause for hospitalisation in our study. Although specific causes of injuries were not explored, it is likely that the injuries may be associated with the high levels of violence in South Africa (28).

There are gender implications that need to be borne in mind when providing adolescent-friendly health services. More males in our study reported drug use, earlier sexual debut, and consultations for condom breakages. Though much has been written about sexual health services for females, our work demonstrates the importance of including sexual health and drug counselling services for adolescent males. The association between male adolescents and drug use has been documented in other studies (29).

Our data reflect the vulnerable nature of adolescents in Soweto: just over three-quarters of 14–19 year olds surveyed reported medium-to-high food insecurity. This reflects prevalent poverty, which is an important social determinant of health (3). Multi-sectoral interventions to address food insecurity are needed on a large scale. Indeed, in a few African studies, conditional cash-transfer systems have been noted to reduce risky behaviours in girls (30–32). The South African government already provides child support grants to about 60% of children below the age of 18 years, and there is evidence to show this money is being used for essential needs (33). However, these grants are not conditional on anything except low parent or guardian income. We recommend removing barriers to accessing social grants for vulnerable youth who are both in and out-of-school, linking vulnerable schools with existing food and nutrition schemes, and re-establishing links between public education and health delivery systems.

Some limitations to this study should be noted. Measures were self-reported to an interviewer and therefore possibly influenced by recall and social desirability bias. We did not collect information on injury types. Neither did we explore what exactly participant concerns about HIV were as a reason for seeking healthcare. Voluntary response bias, defined as self-selected volunteers, may have occurred during the sampling process where adolescents were brought into a youth-friendly clinic for interviews from non-healthcare settings. The statistical analysis did not focus on causal inference as this was a cross-sectional non-random sample. Though not generalizable, our findings have relevance to other settings in sub-Saharan Africa where there is need for more research and programming in supporting health-seeking behaviours among adolescents.

## Conclusions

Despite the existence of general, reproductive healthcare and counselling services within the South African healthcare system, adolescents reported a gap between their needs and the availability of these services; this may reflect unacceptability and perhaps inaccessibility of these services. One recommendation to address this is to revive schools-based health facilities in South Africa by incorporating gender-appropriate services to promote health and treat disease. Last, adolescent voices within health services planning are required to ensure adolescent-friendly, relevant, and appropriate health service delivery.

## References

[1] Statistics South Africa (2011). Mid year population estimates. http://www.statssa.gov.za/publications/P0302/P03022011.pdf.

[2] Barker G (2007). Adolescents, social support and help seeking behaviour. http://whqlibdoc.who.int/publications/2007/9789241595711_eng.pdf.

[3] National Youth Commission (2009). National youth policy 2009–2014.

[4] WHO (2014). Adolescent health epidemiology. http://www.who.int/maternal_child_adolescent/epidemiology/adolescence/en/.

[5] Cooper D, Dickson K, Blanchard K, Cullingworth L, Mavimbela N, von Mollendorf C (2005). Medical abortion: the possibilities for introduction in the public sector in South Africa. Reprod Health Matters.

[6] Richter LM, Norris SA, Ginsburg C (2006). The silent truth of teenage pregnancies–Birth to Twenty cohort’s next generation. S Afr Med J.

[7] Dunkle KL, Jewkes RK, Brown HC, Yoshihama M, Gray GE, McIntyre JA (2004). Prevalence and patterns of gender-based violence and revictimization among women attending antenatal clinics in Soweto, South Africa. Am J Epidemiol.

[8] van Rooyen H, McGrath N, Chirowodza A, Joseph P, Fiamma A, Gray G (2013). Mobile VCT: reaching men and young people in urban and rural South African pilot studies (NIMH Project Accept, HPTN 043). AIDS Behav.

[9] Katz IT, Nkala B, Dietrich J, Wallace M, Bekker LG, Pollenz K (2013). A qualitative analysis of factors influencing HPV vaccine uptake in Soweto, South Africa among adolescents and their caregivers. PLoS One.

[10] Seedat M, Van Niekerk A, Jewkes R, Suffla S, Ratele K (2009). Violence and injuries in South Africa: prioritising an agenda for prevention. Lancet.

[11] Dickson-Tetteh K, Pettifor A, Moleko W (2001). Working with public sector clinics to provide adolescent-friendly services in South Africa. Reprod Health Matters.

[12] LoveLife Youth-friendly HCT. http://www.lovelife.org.za/corporate/files/4113/3848/5198/yfs_email_document.indd_Final_Final.pdf.

[13] City of Johannesburg (2011). Census 2011 – main place “Soweto”. http://census2011.adrianfrith.com/place/798026.

[14] Statistics South Africa (2012). Census 2011 municipal report Gauteng. http://www.statssa.gov.za/Census2011/Products/GP_Municipal_Report.pdf.

[15] Coates J, Swindale S, Bilinsky P (2007). Household Food Insecurity Access Scale (HFIAS) for measurement of food access: indicator guide.

[16] Radloff LS (1977). The CES-D scale a self-report depression scale for research in the general population. Appl Psychol Meas.

[17] Chabrol H, Montovany A, Chouicha K, Duconge E (2002). [Study of the CES-D on a sample of 1,953 adolescent students]. Encephale.

[18] SAS Institute Inc (2009). SAS/STAT^®^ 9.2 user’s guide.

[19] Kisker EE, Brown RS (1996). Do school-based health centers improve adolescents’ access to health care, health status, and risk-taking behavior?. J Adolesc Health.

[20] Richter MS, Mfolo V (2006). The perception of South African adolescents regarding primary health care services. Sci World J.

[21] Health Systems Trust (2012). National health care facilities baseline audit. http://www.hst.org.za/publications/national-health-care-facilities-baseline-audit-national-summary-report.

[22] Hampanda K, Ybarra M, Bull S (2014). Perceptions of health care services and HIV-related health-seeking behavior among Uganda adolescents. AIDS Care.

[23] Mmari KN, Oseni O, Fatusi AO (2010). STI treatment-seeking behaviors among youth in Nigeria: are there gender differences?. Int Perspect Sex Reprod Health.

[24] Mmari K, Blum R, Sonenstein F, Marshall B, Brahmbhatt H, Venables E (2014). Adolescents’ perceptions of health from disadvantaged urban communities: findings from the WAVE study. Soc Sci Med.

[25] Plüddemann A, Flisher AJ, McKetin R, Parry C, Lombard C (2010). Methamphetamine use, aggressive behavior and other mental health issues among high-school students in Cape Town, South Africa. Drug Alcohol Depend.

[26] Ramirez-Avila L, Regan S, Giddy J, Chetty S, Ross D, Katz JN (2012). Depressive symptoms and their impact on health-seeking behaviors in newly-diagnosed HIV-infected patients in Durban, South Africa. AIDS Behav.

[27] Rosen EU (1988). Adolescent health problems–can paediatricians in SA cope?. S Afr Med J.

[28] Mayosi BM, Lawn JE, van Niekerk A, Bradshaw D, Abdool Karim SS, Coovadia HM (2012). Health in South Africa: changes and challenges since 2009. Lancet.

[29] Madu SN, Matla MP (2003). Illicit drug use, cigarette smoking and alcohol drinking behaviour among a sample of high school adolescents in the Pietersburg area of the Northern Province, South Africa. J Adolesc.

[30] Campbell C, Scott K, Nhamo M, Nyamukapa C, Madanhire C, Skovdal M (2013). Social capital and HIV competent communities: the role of community groups in managing HIV/AIDS in rural Zimbabwe. AIDS Care.

[31] Handa S, Pettifor A, Thirumurthy H, Halpern C (2012). Effect of a national social cash transfer program on HIV risk behavior in Kenya. http://www.iasociety.org/Abstracts/A200747618.aspx.

[32] Skovdal M, Robertson L, Mushati P, Nyamukapa C, Sherr L, Gregson S (2012). Community-led cash transfers in Zimbabwe: pathways for buy-in and improved child health and development. http://iasociety.org/Abstracts/A200744209.aspx.

[33] Budlender D, Woolard I (2006). The impact of the South African child support and old age grants on children’s schooling.

